# SPDC‐HG: An accelerator of genomic hybrid breeding in maize

**DOI:** 10.1111/pbi.70011

**Published:** 2025-02-27

**Authors:** Zhenliang Zhang, Xin Wang, Yuxiang Zhang, Kai Zhou, Guangning Yu, Wenyan Yang, Furong Li, Xiusheng Guan, Xuecai Zhang, Zefeng Yang, Chenwu Xu, Yang Xu

**Affiliations:** ^1^ Key Laboratory of Plant Functional Genomics of the Ministry of Education/Jiangsu Key Laboratory of Crop Genomics and Molecular Breeding/Jiangsu Co‐Innovation Center for Modern Production Technology of Grain Crops, College of Agriculture Yangzhou University Yangzhou Jiangsu China; ^2^ Jiangsu Yanjiang Institute of Agricultural Sciences Nantong China; ^3^ College of Information Engineering Yangzhou University Yangzhou Jiangsu China; ^4^ International Maize and Wheat Improvement Center (CIMMYT) Texcoco México

**Keywords:** maize, sparse partial diallel cross design, general combining ability, genomic selection, hybrid breeding

## Abstract

Integrating multiple modern breeding techniques in maize has always been challenging. This study aimed to address this issue by applying a flexible sparse partial diallel cross design composed of 945 maize hybrids derived from 266 inbred lines across different heterotic groups. The research integrated genome‐wide association studies, genomic selection and genomic evaluation of parental inbred lines to accelerate the breeding process for developing single‐cross hybrids. Significant associations were identified for 7–25 stable single nucleotide polymorphisms (SNPs) associated with the general combining abilities (GCAs) of nine yield‐related traits. Using the maizeGDB and NCBI databases, 264 candidate genes were screened and functionally annotated based on significant SNPs detected by at least three statistical methods. The marker set developed from these GCA SNPs significantly improved the prediction accuracy of hybrids across all traits. The GCA estimates of the inbred lines involved in the top 100 and bottom 100 hybrids consistently ranked at the top and bottom, thereby confirming the accuracy of the predictions. Furthermore, the top 100 crosses selected using BayesB, GBLUP and LASSO showed a 105.4–108.6% increase in average ear weight compared to the bottom 100 crosses in field validation, demonstrating strong selection gains. Notably, amongst the top 100 hybrids, A017/A037 and A037/A169, each containing six superior genotypes were registered as Suyu 161 and Tongyu 1701, respectively, by the National Crop Variety Approval Committee in China. These results highlight the effectiveness of genomic selection and provide valuable insights for advancing genomic hybrid breeding in maize.

## Introduction

Maize is an essential crop for food, animal feed and bioenergy production, and is already the leading cereal worldwide in terms of production (Erenstein *et al*., 2022). Hybrid breeding remains the most effective strategy for improving maize yield potential (Li *et al*., 2022a). Since the 1960s, the development of single‐cross hybrids by crossing parental lines from different heterotic groups (HGs) has revolutionized maize breeding (Li *et al*., 2023). Maize breeding involves two essential steps: developing inbred lines with high general combining ability (GCA) and selecting suitable parent inbred lines to create elite hybrids.

The GCA concept, which was initially proposed by Sprague and Tatum (1942), pertains to the overall performance of a parent line across all hybrid combinations. GCA is predominantly influenced by additive genetic effects that can be directly transmitted from parents to offspring (Chen *et al*., 2019). High GCA effects imply that the line possesses high breeding value for specific traits, making it a valuable candidate for breeding programmes. The advent of double‐haploid technology has facilitated the annual generation of numerous inbred lines, but GCA assessment remains a bottleneck in maize breeding. Although GCA can be readily estimated using a complete diallel cross design or North Carolina Design II (NCII), these methods are labour‐intensive and time‐consuming when dealing with a large number of inbred lines. With the development of genomic technologies, there is growing interest in integrating molecular markers to enhance GCA evaluation and prediction. In maize, the GCAs for seven traits were predicted using 56,110 single nucleotide polymorphisms (SNPs) derived from 285 diverse inbred lines crossed with two testers. The prediction accuracies ranged from 0.72 to 0.81 (Riedelsheimer *et al*., 2012). The prediction abilities of GCA for grain yield varied from 0.49 to 0.55 across three maize line‐by‐tester trials (Zhang *et al*., 2022). The GCA classification of eight inbred maize lines, estimated using genomic data, was found to be consistent with phenotypic evaluations (Vélez‐Torres *et al*., 2018). Additionally, a few quantitative trait loci (QTL) associated with the GCA of maize agronomic traits have been identified through linkage mapping, aiding in understanding the genetic basis of GCA. However, current GCA studies are constrained by a limited number of testers, which reduces the accuracy of GCA estimates. Therefore, it is imperative to develop a novel scheme to evaluate and dissect GCA.

Identifying the optimal crosses between the selected lines is another significant challenge in hybrid breeding. It is virtually impossible to assess all the potential crosses in the field because of limited resources. Genomic selection (GS) is a promising approach to address these challenges. It uses genome‐wide markers to predict the genetic value of individuals before phenotyping (Alemu *et al*., 2024; Meuwissen *et al*., 2001). GS is particularly beneficial for hybrid breeding because it can be used to infer the genotypes of hybrids using those of their parents, thereby accelerating the breeding process and reducing costs. Several recent studies have used GS to predict the performance of maize hybrids, demonstrating moderate‐to‐high predictability for various traits. The prediction accuracies for grain yield ranged from 0.28 to 0.77, whilst those for plant height (PH) ranged from 0.53 to 0.91, as determined through cross‐validation across different numbers of tested parents and GS models (Kadam *et al*., 2016). The mean prediction accuracies for nine yield‐related traits varied from 0.46 to 0.89, based on an analysis of 285 hybrids derived from a partial diallel cross design (Luo *et al*., 2023). The average predictive abilities for ten agronomic traits in 490 hybrids derived from crosses between 119 inbred lines ranged from 0.39 to 0.79. Notably, when the top 44 hybrids were selected based on these predictions, a 6% increase in grain yield was achieved compared to a commercial hybrid (Li *et al*., 2020). Although these studies are vital for evaluating GS models, it is noteworthy that high prediction accuracy does not guarantee the superiority of selected varieties. Breeders are more interested in the actual performance of the top varieties identified through GS. Therefore, further validation is required to bridge the gap between genomic prediction (GP) and practical breeding applications.

The simultaneous integration of multiple modern breeding techniques has always been challenging in maize breeding. In this study, we present a new genetic mating design based on a sparse partial diallel cross (SPDC) between different HGs, abbreviated as SPDC‐HG. Based on this design, we used 266 inbred lines from five different HGs to create 945 hybrids, referred to as the SPDC‐HG population. Using this population, we performed a joint analysis of a genome‐wide association study (GWAS), GS and genomic evaluation of parental inbred lines to help accelerate breeding progress for producing superior hybrids. The aim of this study was to propose a genetic mating design (SPDC‐HG) and its corresponding joint analysis scheme. Our specific research included three parts: (a) accurately estimating the GCA of nine yield‐related traits for 266 inbred lines using genomic information; (b) exploring the genetic basis of GCA and clarifying the relationship between GCA and hybrid performance and (c) using field trials to validate the top‐selection gain of GS for maize hybrids, compare the top‐selection gain of three popular GS methods and select the hybrid variety with the greatest breeding potential for maize.

## Results

### Performance of the SPDC‐HG population

**Figure 1:**
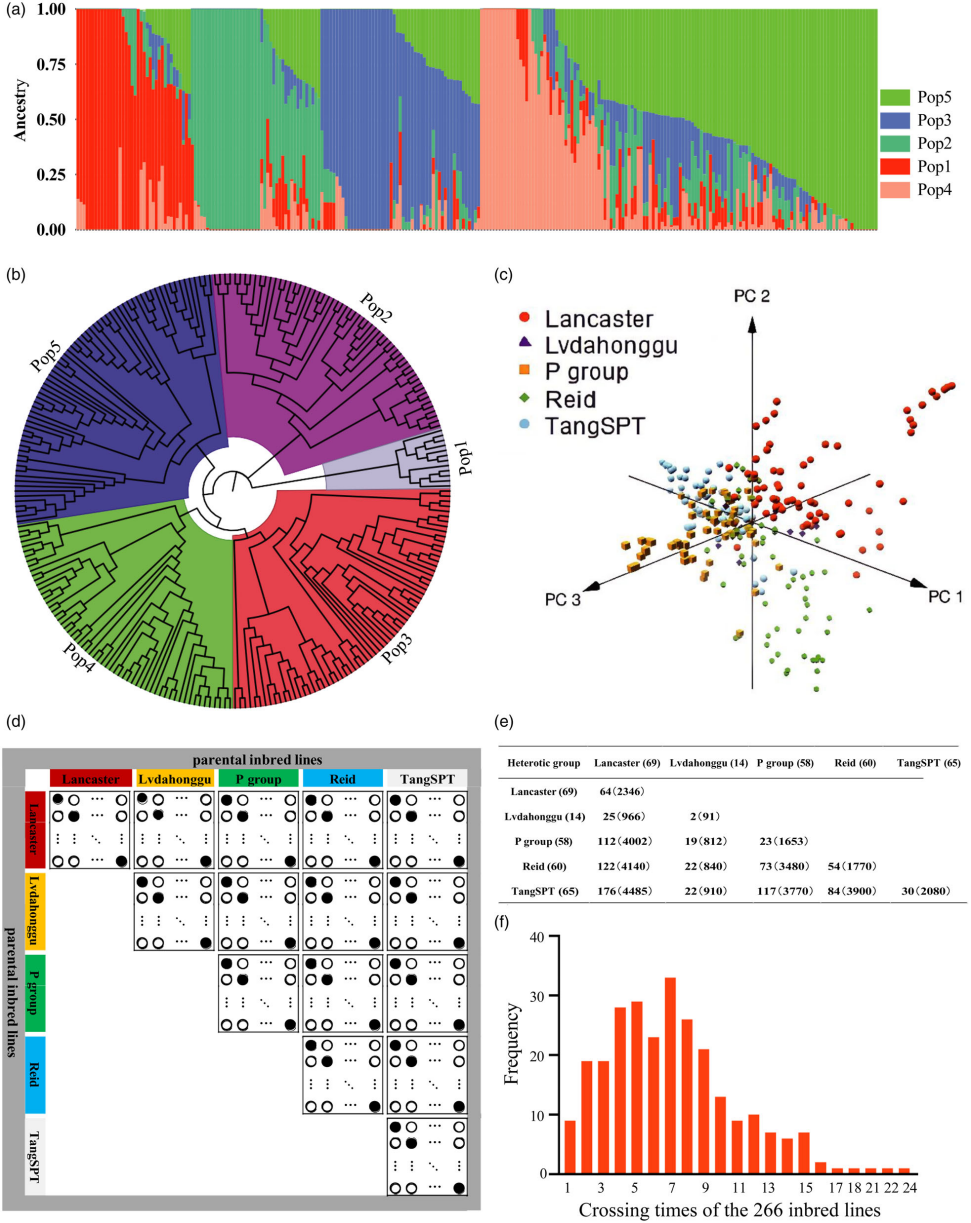
Development of the SPDC‐HG population. (a) Population structure of 266 inbred lines from Admixture. (b) Neighbor‐joining (NJ) tree of the 266 inbred lines. (c) PCA plot of the first three principal components for the 266 inbred lines. (d) Schematic diagram of the sparse partial diallel cross (SPDC) between different HGs (the solid circle represents the hybrid to be crossed, whilst the hollow circle represents the hybrid to be predicted). (e) Actual crossing outcomes between different heterotic groups in the SPDC‐HG population: numbers outside the brackets represent actual crosses, whilst numbers within brackets represent theoretical crosses. (f) Frequency distribution of the crossing times for the 266 parental lines.

The population structure of 266 inbred lines was investigated using Admixture, neighbor‐joining tree and pedigree information (Figure 1a,b). Through comprehensive analysis, the 266 inbred lines were categorized into five HGs: Lvdahonggu, TangSPT, Reid, Lancaster and P group, consisting of 14, 65, 60, 69 and 58 lines, respectively (Table S1). These groups accounted for 5.26%, 24.44%, 22.56%, 25.94% and 21.80% of the total number of inbred lines, respectively. Moreover, these groups have been reported in previous studies (Lu *et al*., 2009; Wang *et al*., 2008) and have been widely utilized in maize hybrid breeding programmes in China over recent decades. The population structure analysis indicated that the 266 selected inbred lines possessed a broad genetic basis. Furthermore, principal component analysis (PCA) was employed to illustrate the division results of the five HGs (Figure 1c). The first three principal components effectively distinguished amongst the five HGs, confirming the reliability of their classification.

A SPDC‐HG population was developed based on a sparse partial diallel cross between different HGs (Figure 1d). Owing to the complex environmental factors and unexpected flowering periods of these inbred lines, the SPDC‐HG population was not fully crossed according to the SPDC‐HG design. Nevertheless, a total of 945 hybrids were generated for the training set, excluding reciprocals. This set included 772 hybrids crossed between two parental lines from different HGs and 173 hybrids crossed between two parental lines within the same HG (Figure 1e). Each parental line participated in 1–24 crosses (Figure 1f), with an average of seven crosses per inbred line. A detailed crossing scheme is presented in Table S2.

We investigated nine yield‐related traits in the SPDC‐HG population in the two environments. We conducted a descriptive statistical analysis of various heterotic patterns for each trait (Figure S1; Table S3). Variance analysis revealed significant differences (*P* < 0.01) between genotypes and environments (Table S3), indicating that the yield‐related traits are influenced by genetic factors, environmental conditions and the interaction between genetics and environment. To minimize the impact of environmental factors on genetic effects, the best linear unbiased predictor (BLUP) values of the nine traits were utilized for further analysis (Table S4). For ear weight (EW) and ear grain weight (EGW), the heterotic patterns of Lvdahonggu × P and P × Reid exhibited the highest mean values. Additionally, these two patterns demonstrated greater PH, kernel number per row (KNR), ear row number (ERN), ear length (EL), ear diameter (ED), relatively lower ear height (EH) and cob diameter (CD). These results indicated that these two patterns represented optimal heterotic patterns that could be further utilized in maize breeding.

### GS for maize hybrids and field validation

Based on the SPDC‐HG population, we evaluated prediction accuracies of three GS methods (BayesB, GBLUP and LASSO) across the nine traits using replicated fivefold cross‐validation (Figure S2). The prediction accuracies varied from 0.473 to 0.795 across different traits and models. Amongst the three methods, no significant differences (*P* < 0.05) were observed in the prediction accuracies for the traits of EW, EGW and EH. However, GBLUP and BayesB exhibited slightly higher prediction accuracies for ERN, ED, CD and PH compared to LASSO. Notably, ERN exhibited the highest prediction accuracy when utilizing BayesB, GBLUP and LASSO, with values of 0.794, 0.795 and 0.790, respectively. In contrast, KNR demonstrated the lowest prediction accuracy across the three methods, with values of 0.485, 0.479 and 0.473 for BayesB, GBLUP and LASSO, respectively. The prediction accuracies for the three methods ranged from 0.679 to 0.683 for EW and from 0.652 to 0.653 for EGW.

**Figure 2:**
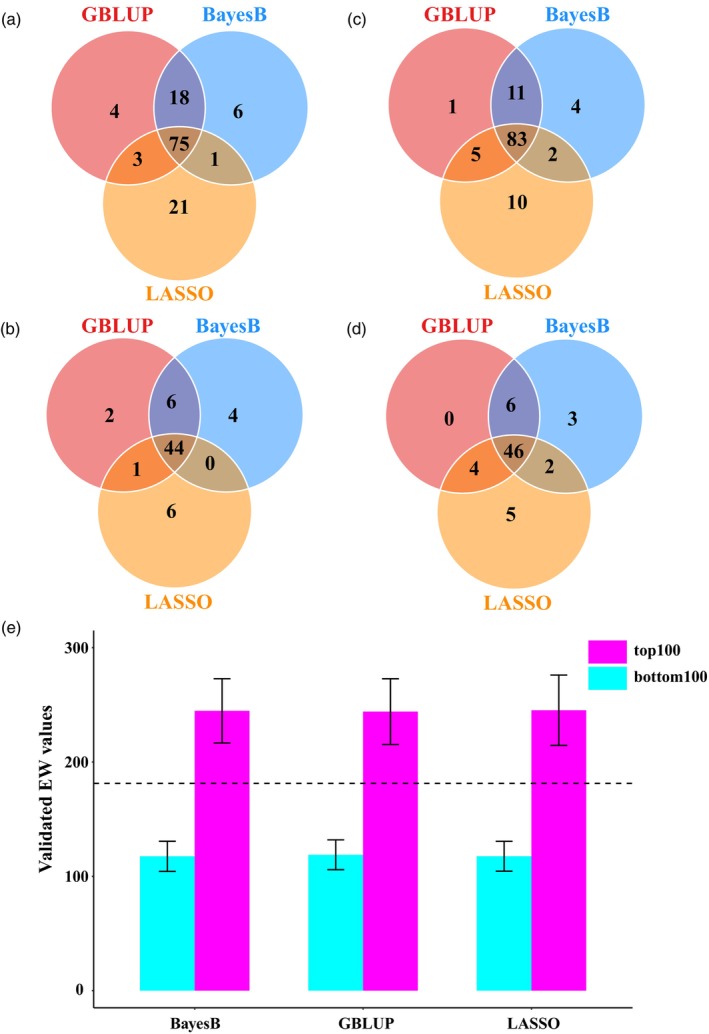
Top‐selection gain of three GS methods validated by field trials. (a–d) Venn diagrams of selected hybrids using GBLUP, BayesB and LASSO. (a) Venn diagram of the top 100 hybrids selected by the three GS methods. (b) Venn diagram of the top 63 hybrids successfully validated in the field. (c) Venn diagram of the bottom 100 hybrids selected by the three GS methods. (d) Venn diagram of the bottom 66 hybrids successfully validated in the field. (e) EW values of the top and bottom hybrids successfully validated in the field (mean ± SD). The horizontal dashed line (181.014) represents the mean of the hybrids validated in the field.

For 266 inbred lines, there were a total of 35,245 potential hybrids, calculated with 266 × (266−1)/2. The predicted EW phenotypes of these hybrids were derived from the SPDC‐HG population (Table S5). The top 100 hybrids with the highest predicted EW values and the bottom 100 hybrids with the lowest predicted EW values were selected using each of the three prediction methods and subsequently cultivated in Yangzhou, China. The selection results demonstrated a high level of consistency across all three GS methods (Figure 2a–d). There were 244 individuals in the union of the varieties selected from the three methods, of which 129 hybrids were successfully validated in the field, including the top 63 and bottom 66 hybrids (Hybrid_Set1, Table S6). The average validated EW values of the top 100 crosses selected using BayesB, GBLUP and LASSO were 108.3%, 105.4% and 108.6% higher than those of the bottom 100 crosses, respectively (Figure 2e). Compared to the mean of the hybrids validated in the field, the average EW of the top 100 hybrids increased by 35.2%, 34.8% and 35.5% using the BayesB, GBLUP and LASSO prediction models, respectively. Conversely, the average EW of the bottom 100 hybrids decreased by 35.1%, 34.3% and 35.1% for the BayesB, GBLUP and LASSO prediction models, respectively. The mean EW of the check variety ‘Suyu29’ was 247.5 g, and 22 hybrids with field validation exhibited an EW higher than that of the check variety, with 17 hybrids surpassing the check variety by more than 5%. Collectively, these results demonstrate the potential applications of GS in maize breeding.

### Accurate estimation of GCA

**Figure 3:**
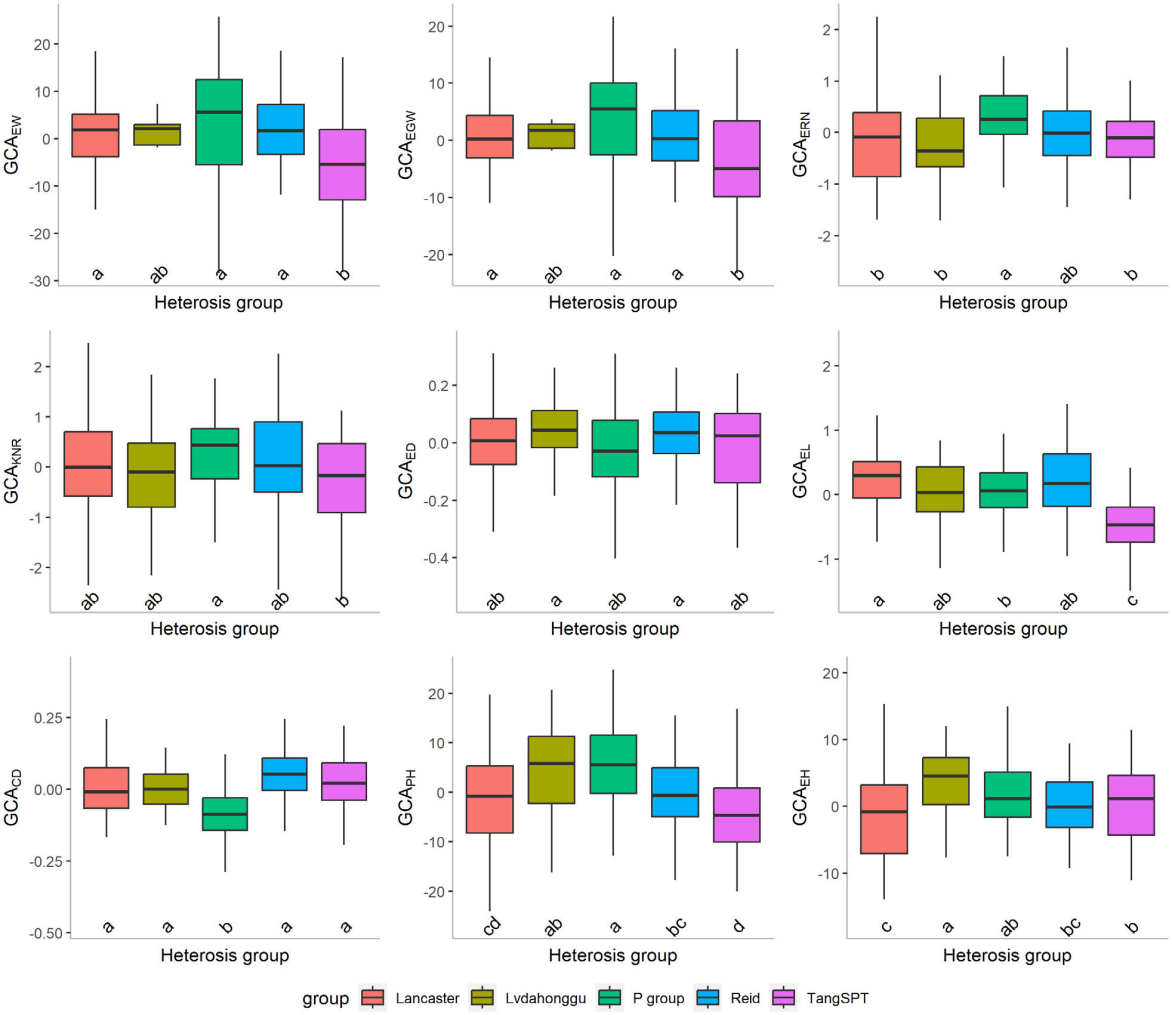
Phenotypic distributions of GCAs for nine yield‐related traits categorized according to different heterotic groups.

With advancements in modern molecular biology, breeders can accurately estimate GCA using GP. In this study, we used genomic information and a linear mixed model to predict the phenotypes of all possible hybrids using the SPDC‐HG population, allowing precise estimation of the GCA values for 266 inbred lines (Table S7). The GCA values for each trait were normally distributed, with the averages centred around zero and a wide range of variation (Table S8). Significant differences (*P* < 0.05) were observed amongst the various HGs for each trait (Table S8; Figure 3). The P group showed the highest values for GCA_EW_, GCA_EGW_, GCA_ERN_, GCA_KNR_ and GCA_PH_, indicating strong breeding potential for yield and ear traits. In the Reid group, four traits, GCA_EW_, GCA_EGW_, GCA_ERN_ and GCA_KNR_, ranked just below those of the P group, demonstrating relatively higher GCA values. Within this group, the GCA values for ED and CD were highest, whereas those for GCA_PH_ and GCA_EH_ were negative and comparatively lower. This suggests that the Reid group has strong breeding potential for yield and ear type, whilst effectively reducing PH and EH. The Lancaster group exhibited the highest GCA_EL_ and lowest GCA_EH_ values, whereas the GCA values for the other traits were relatively moderate. The Lvdahonggu group exhibited the highest values for GCA_ED_ and GCA_EH_. In contrast, the GCA values for the other traits were comparatively low or negative, suggesting that this group has the potential to enhance ED, PH and EH but lacks the potential to increase yield. The TangSPT group exhibited the lowest values of GCA_EW_, GCA_EGW_, GCA_KNR_, GCA_EL_, GCA_CD_ and GCA_PH_. During the breeding process, elite inbred lines from the P group can be used for backcrossing and breeding enhancement.

To investigate the prediction accuracy of GCA, we conducted cross‐validation analyses for GCAs of nine traits using BayesB, GBLUP and LASSO (Figure S3). By comparing the prediction accuracies (*r*) amongst the nine traits and three methods (BayesB, GBLUP and LASSO), we observed that the highest prediction accuracies of 0.827, 0.833 and 0.778 were achieved for GCA_CD_, respectively. By contrast, the lowest prediction accuracies of 0.644, 0.658 and 0.522 were recorded for GCA_KNR_. Notably, GBLUP yielded the best results for all GCA traits. For GCA_KNR_, there were significant differences in prediction accuracies amongst the three GS methods. GBLUP demonstrated the highest prediction accuracy, followed by BayesB, whilst LASSO exhibited the lowest. For the GCA of the other traits, there was no obvious difference between GBLUP and BayesB in terms of prediction accuracy, and the two methods provided better prediction results than LASSO. For instance, for GCA_EW_, the prediction accuracies of GBLUP and BayesB were 0.734 and 0.730, respectively, which were 8.1% and 7.5% higher than those of LASSO.

### GWAS of yield‐related traits and their corresponding GCA

**Figure 4:**
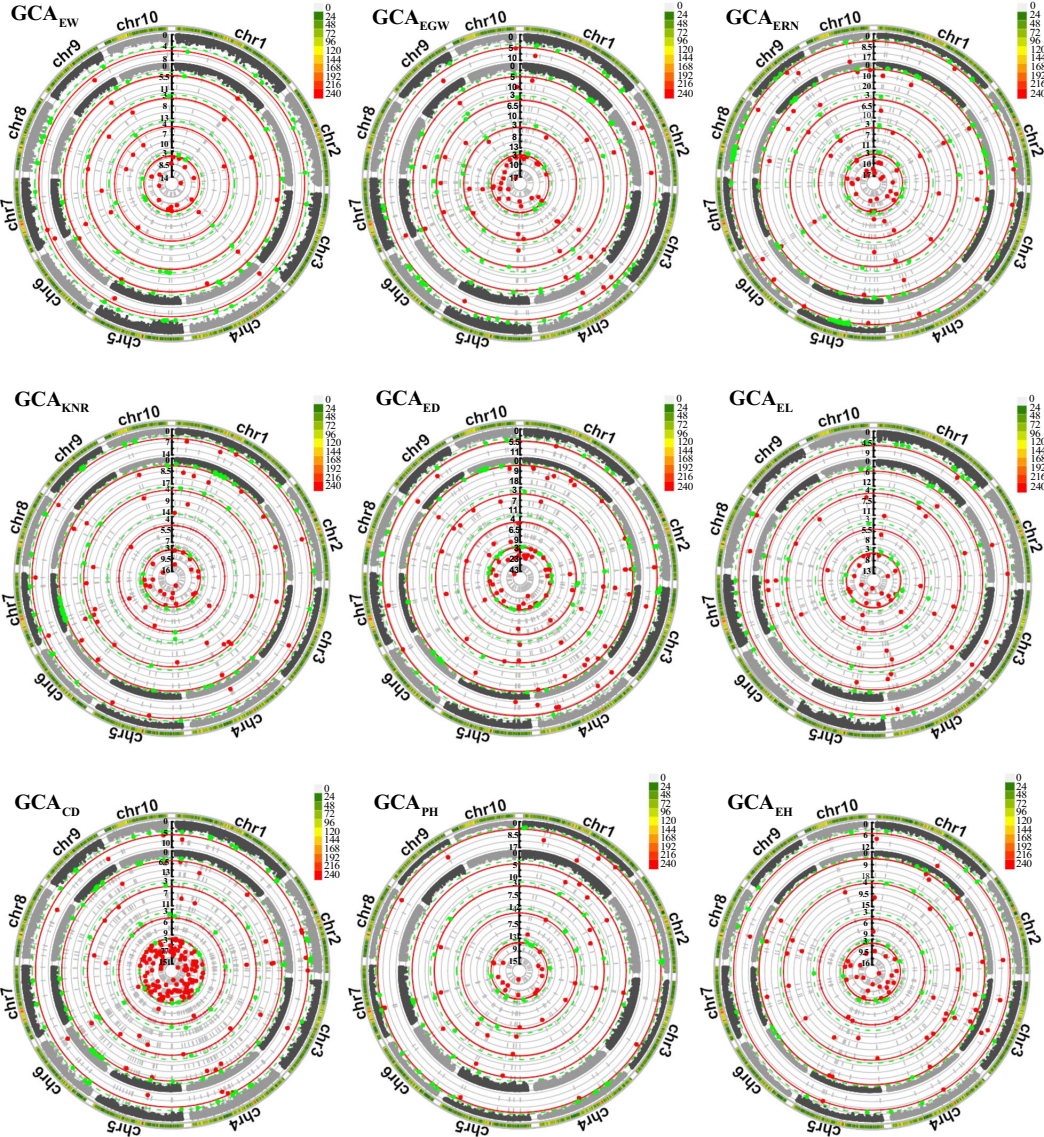
Manhattan plots of GCAs for nine yield‐related traits using five methods. The outermost layer shows the SNP marker density across the chromosomes, with five y‐axes corresponding to the results obtained from Blink, FarmCPU, FASTmrMLM, FASTmrEMMA and ISIS EM‐BLASSO, respectively (from outside to inside). The red line indicates the threshold of −log_10_ (1/108541) = 5.036, and the green dotted line indicates the default LOD threshold of 3.

GWAS were conducted on yield‐related traits and their corresponding GCA values using five multi‐locus methods, including Blink, FarmCPU, FASTmrMLM, FASTmrEMMA and ISIS EM‐BLASSO (Figure 4; Figure S4). A total of 630 significant SNPs were identified for GCAs (Table S9; Figure 4). The following significant SNPs were identified: 44 for GCA_EW_, 64 for GCA_EGW_, 69 for GCA_ERN_, 65 for GCA_KNR_, 102 for GCA_ED_, 60 for GCA_EL_, 168 for GCA_CD_, 54 for GCA_PH_ and 64 for GCA_EH_. Additionally, 299 significant SNPs were identified for yield‐related traits (Table S10; Figure S4), with the following significant SNPs identified: 35 for EW, 27 for EGW, 39 for ERN, 26 for KNR, 44 for ED, 36 for EL, 45 for CD, 40 for PH and 34 for EH. To minimize the influence of statistical methods on the association analysis, the SNPs identified by at least two different statistical methods were considered stable SNPs (Tables S11, S12). The results indicated that 7, 16, 13, 14, 14, 10, 25, 13 and 15 stable SNPs were significantly associated with GCA_EW_, GCA_EGW_, GCA_ERN_, GCA_KNR_, GCA_ED_, GCA_EL_, GCA_CD_, GCA_PH_ and GCA_EH_, respectively. Additionally, 6, 5, 9, 1, 4, 5, 16, 3 and 6 stable SNPs were significantly associated with EW, EGW, ERN, KNR, ED, EL, CD, PH and EH, respectively.

The linkage disequilibrium (LD) analysis results of the association panel indicated that when *r* ^*2*^ was > 0.2, LD decay with physical distance in our association panel was about 210 kb (Figure S5). Related candidate genes within the LD range of significant SNPs were searched using the maizeGDB and NCBI databases. The maize genome B73 RefGen_v4 served as the reference genome for candidate gene analyses. A total of 264 candidate genes (Table S13) were identified and functionally annotated based on significant SNPs detected by at least three GWAS methods. These candidate genes were primarily involved in cellular metabolism, endosperm development, photosynthetic metabolic pathways, responses to abiotic stress, plant morphogenesis, root growth and development, auxin homeostasis and other physiological and biochemical processes. *Zm00001d018827* encodes a glutaredoxin protein (GRX), which is involved in antioxidant defense, with some CC‐type GRXs playing a role in stress responses (Couturier *et al*., 2015; Gutsche *et al*., 2015). *Zm00001d037596* encodes the RING/U‐box superfamily protein that modulates auxin signaling output (Feraru *et al*., 2022). *Zm00001d037849* encodes cytochrome P450 superfamily protein, which plays a role in the phenylpropanoid pathway and is involved in the regulation of flavonoid and lignin biosynthesis in plants (Hou *et al*., 2023). *Zm00001d006621* (*ZmCPK37*), encoding a calcium‐dependent protein kinase, has the potential to enhance drought tolerance in maize and mitigate yield loss under drought conditions (Li *et al*., 2022b). This gene is preferentially expressed in the guard cells of maize and plays a crucial role in stomatal closure under drought‐stress conditions. *Zm00001d053338*, which encodes an F‐box/kelch‐repeat protein (Jia *et al*., 2013; Qin *et al*., 2021), is homologous to the F‐box protein gene *LOC Os02g15950.1*, which regulates panicle size and grain weight in rice (Li *et al*., 2011). *Zm00001d012544* (*ZmMYB33‐1*) has been identified as a novel genic male‐sterility gene in maize through DNA sequencing, phenotypic analysis and cytological examinations (Jiang *et al*., 2021).

**Figure 5:**
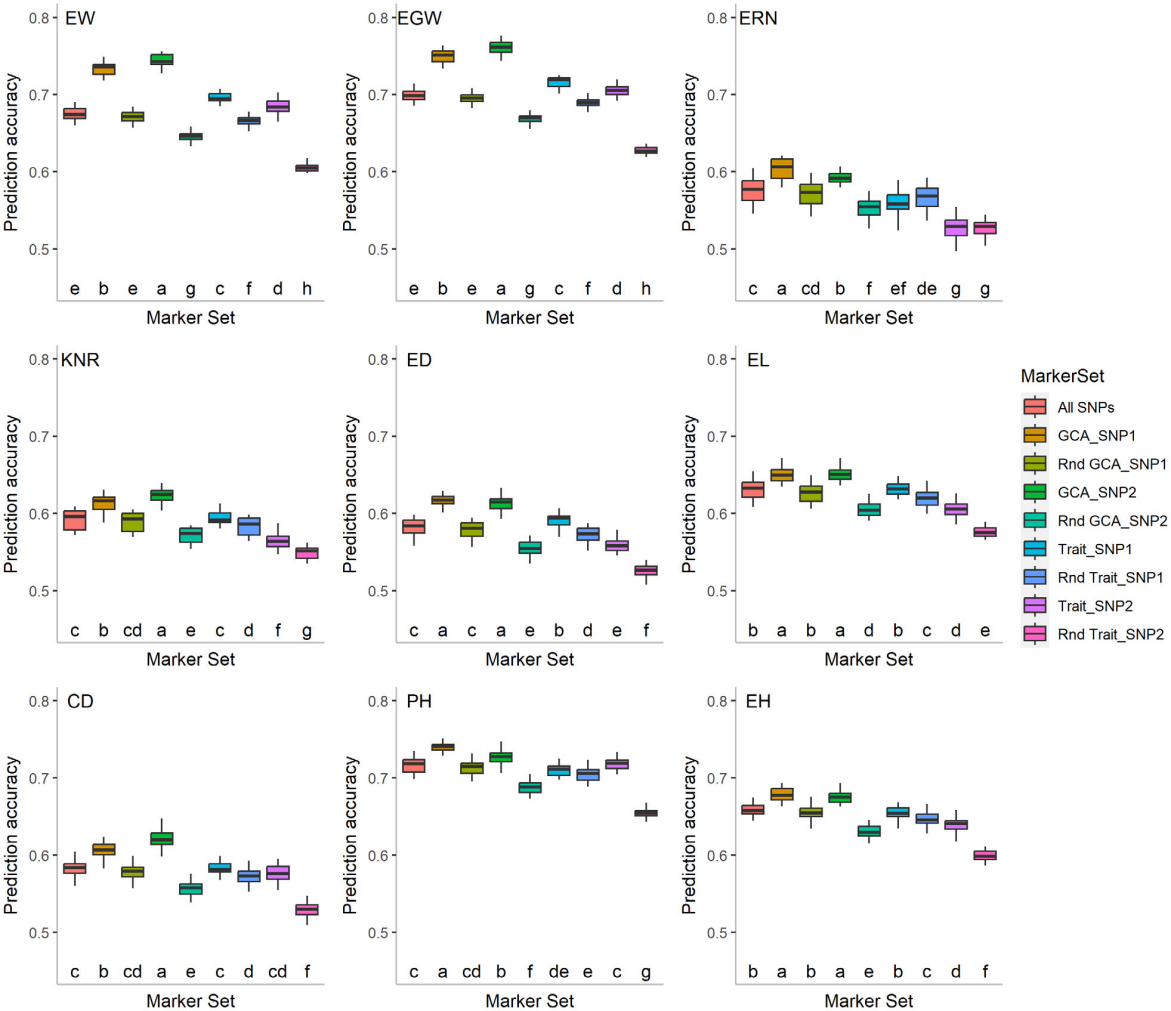
Prediction accuracies of nine yield‐related traits in maize hybrids using nine different SNP marker datasets. All SNPs represent all SNP markers; GCA_SNP1 includes significant GCA SNPs detected by at least one statistical method, whilst Rnd GCA_SNP1 is a randomly selected set of SNPs equivalent in size to GCA_SNP1; GCA_SNP2 consists of significant GCA SNPs detected by at least two statistical methods, and Rnd GCA_SNP2 is a randomly selected set of SNPs of the same size; Trait_SNP1 includes significant SNPs for the trait per se detected by at least one statistical method, whilst Rnd Trait_SNP1 is a randomly selected set matching the number of Trait_SNP1; Trait_SNP2 includes significant SNPs for the trait per se detected by at least two statistical methods, and Rnd Trait_SNP2 is a randomly selected set of SNPs of the same size.

To further analyze the role of these associated SNPs in hybrid prediction, we selected 187 inbred lines from the 266 inbred lines to construct a new hybrid population. A total of 259 hybrids (Hybrid_Set2, Table S14) were successfully crossed to verify the prediction accuracies of the nine marker sets, which comprised various types of SNPs (Figure 5). The marker set All SNPs encompasses all SNP markers. GCA_SNP1 includes significant GCA SNPs detected by at least one statistical method, whilst Rnd GCA_SNP1 is a randomly selected set of SNPs that is equivalent in size to GCA_SNP1. GCA_SNP2 consists of significant GCA SNPs detected by at least two statistical methods, and Rnd GCA_SNP2 is a randomly selected set of SNPs of the same size. Trait_SNP1 includes significant SNPs for the trait per se detected by at least one statistical method, whilst Rnd Trait_SNP1 is a randomly selected set matching the number of Trait_SNP1. Trait_SNP2 includes SNPs for the trait per se detected by at least two methods, and Rnd Trait_SNP2 is a randomly selected set of SNPs of the same size. The results indicated that the prediction accuracies of the nine yield‐related traits in the hybrids, assessed using these marker sets, exhibited a similar tendency. The prediction accuracies of GCA_SNP2 and GCA_SNP1 for the nine traits were significantly higher than those of the other marker sets, whereas the prediction accuracy of Rnd Trait_SNP2 was the lowest. For example, the prediction accuracies of GCA_SNP1 and GCA_SNP2 for EW were 0.733 and 0.744, respectively, representing increases of 8.4% and 10.1% over those of All SNPs. In contrast, compared to All SNPs, the prediction accuracies of Rnd GCA_SNP1 and Rnd GCA_SNP2 decreased slightly by 0.6% and 4.4%, respectively. The prediction accuracies of Trait_SNP1 and Trait_SNP2 were 0.696 and 0.685, respectively, reflecting increases of 3.0% and 1.3% compared to All SNPs. Conversely, Rnd Trait_SNP1 and Rnd Trait_SNP2 exhibited decreases of 1.3% and 10.4%, respectively compared to All SNPs.

The superior genotypes of GCA‐significant SNPs for various traits in inbred lines and hybrids were analyzed in this study. For ED, EGW, EL, ERN, EW and KNR, the corresponding GCA values in the inbred lines and associated hybrid phenotypes exhibited an upward trend with the accumulation of superior genotypes (Figures S6 and S7). For CD, EH and PH, the accumulation of favorable genotypes resulted in a downward trend in the GCA of the corresponding traits in the inbred lines, as well as in the phenotypes of the hybrids. This suggests that the accumulation of superior genotypes may effectively enhance the GCA of ED, EGW, EL, ERN, EW and KNR, along with the phenotypes of the corresponding hybrids. Conversely, the accumulation of superior genotypes may also lead to a reduction in the GCA of CD, PH and EH, as well as in the phenotypes of the corresponding hybrids. Analysis of the significant SNPs associated with GCA in both the inbred lines and hybrids identified five main types (Table S15): (1) Type I, where significant SNPs exhibited significant differences between the two homozygous genotypes in the inbred line and significant differences amongst the three genotypes in the hybrids, and the superior genotypes in the hybrid line were the same as those in the inbred line. This type contained 71 significant SNPs; (2) Type II, where significant differences were observed between the two genotypes in the inbred line and amongst the three genotypes in the hybrid. There were two superior genotypes in the hybrid: one heterozygous and one homozygous, and the homozygous superior genotype was consistent with the homozygous superior genotype in the inbred line. This type contained 34 significant SNPs; (3) Type III contained only two significant SNPs, which exhibited significant differences amongst different genotypes in the inbred line. The superior genotype in the hybrid was the heterozygous genotype; (4) Type IV only contained two significant SNPs, showing significant differences amongst different genotypes in the inbred lines. The superior genotypes in the hybrids were different from the superior genotypes in the inbred lines and (5) Type V contained 18 significant SNPs, and there was no significant difference in performance between different genotypes in inbred lines or hybrids.

### Relationship between EW and the corresponding GCA

**Figure 6:**
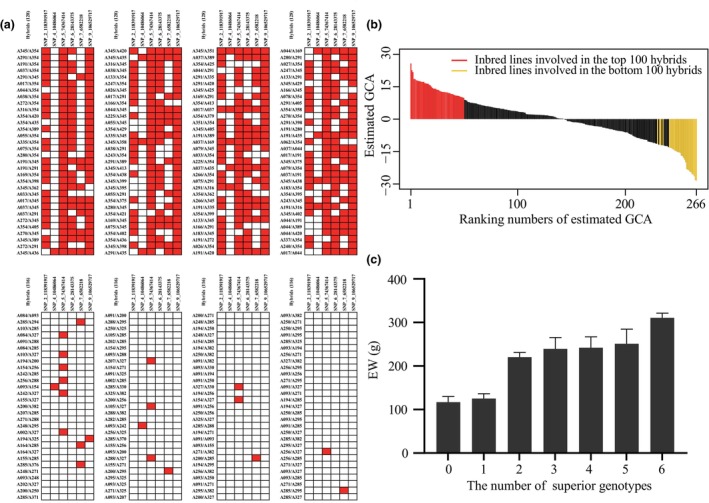
Performance of superior genotypes. (a) Accumulation of superior genotypes in the top 100 and bottom 100 hybrids. (b) Estimated GCA of ear weight (EW) for 266 inbred lines, with red and gold lines representing the inbred lines involved in the top 100 and bottom 100 hybrids, respectively. (c) Variation in EW with the accumulation of the number of superior genotypes in the validation set (the horizontal axis represents the number of superior genotypes (*n*) and the vertical axis denotes ear weight).

As shown in Figure 6b, the inbred lines in the top 100 and bottom 100 hybrids are represented by the red and golden vertical lines, respectively. The estimated GCA_EW_ value of the inbred lines in the top 100 and bottom 100 hybrids always ranked at the top and bottom, respectively, indicating that the estimation of GCA was quite effective. Accurate estimation of the GCA is crucial because crossing parents with higher GCA significantly increases the likelihood of producing top‐performing hybrids. Similarly, crossing parents with lower GCA tends to result in inferior hybrids.

Furthermore, the top 100 hybrids were involved in 50 parental inbred lines, including parents of widely promoted varieties such as DH382, H2671, A489 and S181. Finally, analysis of the accumulation of superior genotypes at significant SNPs associated with GCA_EW_ in the top 100 and bottom 100 hybrids showed that the bottom hybrids selected by GS had little or no accumulation of favorable genotypes, whereas the top hybrids were able to extensively aggregate superior genotypes (Figure 6a). For example, hybrids A345/A351, A017/A037, A037/A169 and A345/A438, from the top 100, each contained six superior genotypes. Notably, A017/A037 and A037/A169 were registered as Suyu 161 and Tongyu 1701, respectively, by the National Crop Variety Approval Committee. Variance analysis showed that the EW of hybrids with different numbers of superior genotypes showed significant differences (*P* < 0.01) and showed an increasing trend with the accumulation of the number of superior genotypes (Figure 6c).

## Discussion

GS is particularly valuable for sparse testing in preliminary yield trials, particularly when a large pool of selected candidates must be narrowed for a more comprehensive evaluation (Wartha and Lorenz, 2021). The accuracy of GS is considerably influenced by the genetic similarity between the training and prediction populations, as well as the genetic diversity within the training population (Wang *et al*., 2018). In this study, we developed a novel genetic mating design scheme, referred to as SPDC‐HG. Based on this design, we used 266 inbred lines from five different HGs to establish a training population for predicting all potential hybrids generated from crosses amongst these inbred lines. This design has fully considered the genetic similarity between the training and test populations and the genetic diversity within the training population. In current genomic hybrid breeding programmes, it is essential for the training population to optimize prediction accuracy whilst considering practical constraints such as the human resources and material resources with the required crosses (Alemu *et al*., 2024; Wang *et al*., 2018). In this study, we used three models (GBLUP, BayesB and LASSO) to evaluate the accuracy of GP based on the training population. The results indicated that these methods exhibited similar performance in terms of prediction accuracy, which was consistent with previous findings (Cui *et al*., 2020; Li *et al*., 2020). In a recent study by Luo *et al*. (2023), the prediction accuracies for the yield performance of hybrids estimated using the GBLUP model were found to be 0.51 in the summer sowing area (SUS) and 0.46 in the spring sowing area (SPS). For the remaining yield‐related traits, prediction accuracies ranged from 0.49 to 0.86 in SUS and from 0.53 to 0.89 in SPS. Another study conducted by Li *et al*. (2020) evaluated the predictive performance of ten agronomic traits in a hybrid maize population using eight different prediction models, demonstrating that the average prediction accuracies ranged from 0.386 to 0.794 across various traits and models. Amongst these, the prediction accuracies of BayesB ranged from 0.418 to 0.794, whilst those of LASSO ranged from 0.386 to 0.791. Specifically, the prediction accuracies of BayesB and LASSO for grain yield were 0.539 and 0.545, respectively. In our study, the prediction accuracies varied from 0.473 to 0.795 across nine traits and three models. These results demonstrate that the SPDC‐HG design could achieve robust prediction accuracy across yield‐related traits.

Breeding programmes utilize recombination to integrate the desired trait combinations, resulting in the development of improved varieties (Bevan *et al*., 2017). The narrow genetic basis of biparental populations may restrict genetic improvements in breeding programmes (Ravelombola *et al*., 2021; Spindel and McCouch, 2016). Although more accurate predictions can be achieved for genetically similar populations, increased relatedness may hinder long‐term genetic gains (Xu *et al*., 2021a). Moreover, predictions based on biparental populations may be inaccurate if unrelated biparental populations with different allelic diversity are used as the training population (Zhang *et al*., 2017). Breeding strategies aimed at harnessing heterosis in maize through the cultivation of single‐cross hybrids primarily rely on diverse, vigorous and productive inbred lines. In this study, a natural population consisting of 266 widely selected inbred lines from five commonly used HGs in modern maize breeding was used as the parental stock for crossing. This population has allowed the accumulation of a much broader range of genetic variations for breeding purposes. Based on the SPDC‐HG design, 945 hybrids in the training population possessed diverse genetic backgrounds. Predicted hybrids should share some degree of relatedness with the training population. In our maize breeding programme, the training population of 945 hybrids could be utilized as a public database to predict other hybrids from seemingly related parents, which has been successfully implemented in rice (Cui *et al*., 2020) and tropical maize lines (Pinho Morais *et al*., 2020).

The enhancement of maize yield relies primarily on the exploitation of heterosis in F1 hybrids. However, the performance of hybrids cannot be accurately predicted based solely on their parental lines; rather, they are significantly influenced by their combining ability, which can be categorized into GCA and specific combining ability (SCA). In the crop breeding process, the contribution of GCA to hybrid performance and the development of inbred lines is greater than that of SCA (Grieder *et al*., 2012; Lu *et al*., 2020). Usually, GCA can be easily estimated using a complete diallel cross design or NCII (Fan *et al*., 2018). However, with the development of breeding programmes, a significant number of inbred lines are produced and the potential combinations of crosses increase rapidly, making these designs time‐ and resource‐intensive. Additionally, unlike trait values obtained through observations and measurements, GCA effects are statistical values that vary depending on the number and variety of tester lines used. The large number of inbred lines generated annually has rendered GCA evaluation a major bottleneck in hybrid maize breeding. The SPDC‐HG design presented in this study is highly promising, offering a viable solution to the challenges faced in hybrid breeding. To explore the impact of incomplete pairing of hybrid combinations according to the SPDC‐HG design, we performed a simulation study. Initially, we inferred the genotypes of all potential 35,245 hybrids using genotype data from 266 inbred lines. Based on this data, we simulated a trait, T7, controlled by 200 QTL with a heritability of 0.7 in the hybrid population. These 200 QTL were evenly distributed across the genome, and their effects followed a gamma distribution. As illustrated in Figure S8, with a fixed number of inbred lines, the number of hybrids used to construct the training set had little effect on accuracy. When the number of inbred lines involved reached 150 and 266, the accuracies of GCA estimation from sample sizes of 1000 and 1500 were nearly the same. In contrast, the number of inbred lines involved had a substantial impact on accuracy. During the construction of the SPDC‐HG population, although some hybrid combinations were not fully crossed due to environmental and flowering factors, we generated 945 hybrids, ensuring that all inbred lines participated in the pairing at least once, with an average of seven participations. The SPDC‐HG design proposed in this study ensures a reliable estimation of GCA.

Although several studies on the application of combining abilities have been published for various crops (Chen *et al*., 2019), only a limited number have focused on the genetic dissection of combining abilities in maize (Giraud *et al*., 2017; Zhou *et al*., 2018). The identification of allelic variations underlying the combining abilities will have significant practical implications for maize breeding. Single‐locus GWAS methods, such as MLM, have been extensively used to identify genetic variants. Although these methods can handle a large number of markers, they focus on one marker at a time, thereby neglecting the joint effects of multiple genetic markers that are essential for understanding complex traits influenced by numerous genes. Additionally, single‐locus methods face challenges related to multiple test corrections for threshold values. To address these issues, multi‐locus GWAS methods have been proposed, as they consider all markers simultaneously and do not necessitate multiple test corrections due to their multi‐locus nature (Wang *et al*., 2016). Analyses of both real and simulated data have demonstrated that multi‐locus models possess higher statistical power and lower false‐positive error rates compared to single‐locus models. For instance, research has shown that FarmCPU enhances statistical power when compared to GLM, MLM, CMLM and FaST‐LMM‐Select across various species, like *Arabidopsis thaliana*, maize and mouse (Liu *et al*., 2016). Moreover, both simulated and real data revealed that FASTmrEMMA outperforms single‐locus approaches such as EMMA, CMLM and ECMLM in terms of detection power and effect estimation (Wen *et al*., 2017). Furthermore, a series of simulation studies indicated that all three multi‐locus methods, including FASTmrEMMA, FarmCPU and LASSO are more powerful than the single‐locus method GEMMA (Xu *et al*., 2018). Here, five multi‐locus models were used to conduct comprehensive GWAS of traits and their corresponding GCA effects. Genomic information was utilized to ensure a reliable estimation of GCA in our SPDC‐HG design, enhancing the accuracy of significant SNPs for GCA compared to previous studies (Chen *et al*., 2019; Townsend *et al*., 2013). To obtain reliable SNPs, only SNPs identified simultaneously using at least three methods were used to predict candidate genes. Selecting optimal marker datasets identified by GWAS for specific traits has proven to be a promising approach for enhancing accuracy in GP (Alemu *et al*., 2023; Anilkumar *et al*., 2023; Kim *et al*., 2022). In this study, we developed a new dataset comprising 259 hybrids to assess the prediction accuracy of marker sets. Our findings suggest that SNPs significantly associated with GCA have considerable potential for hybrid breeding. This is mainly because most SNPs in large marker datasets are phenotypically neutral, with only a relatively small proportion being relevant to specific traits (Al Kalaldeh *et al*., 2019; Weber *et al*., 2023).

Within the GS framework, breeders primarily focus on the field performance of the top‐selected varieties. However, only a limited number of studies have conducted field trials to validate the selection gains of the predicted hybrids in maize (Auinger *et al*., 2021; Li *et al*., 2020). Theoretically, predicting the top population and conducting subsequent field trials is straightforward. However, in practice, directly measuring the gains from top selection is challenging. Because of the vast number of potential hybrids, observing their phenotypes in the field is nearly impossible, resulting in the top population lacking a benchmark for comparison. In this study, the control check variety Suyu29 was utilized as the benchmark. Suyu29, developed by the Jiangsu Academy of Agricultural Sciences, served as the control variety for spring maize experimental programmes in Southeast China (https://www.natesc.org.cn/). In the present study, 22 hybrids underwent field validation and exhibited an EW greater than that of Suyu29 (247.5 g). Amongst these, 17 hybrids surpassed the control variety by >5% in EW. The two hybrid combinations, A017/A037 and A037/A169, exhibited the highest EW in the field. Interestingly, these two hybrid combinations also demonstrated superiority in other traits, such as plant type characteristics, disease resistance and lodging resistance, and have been recognized by the National Crop Variety Approval Committee. This information indicates that GS has significant potential for hybrid maize breeding. The remaining 15 hybrid combinations warrant further investigation in future field experiments.

**Figure 7:**
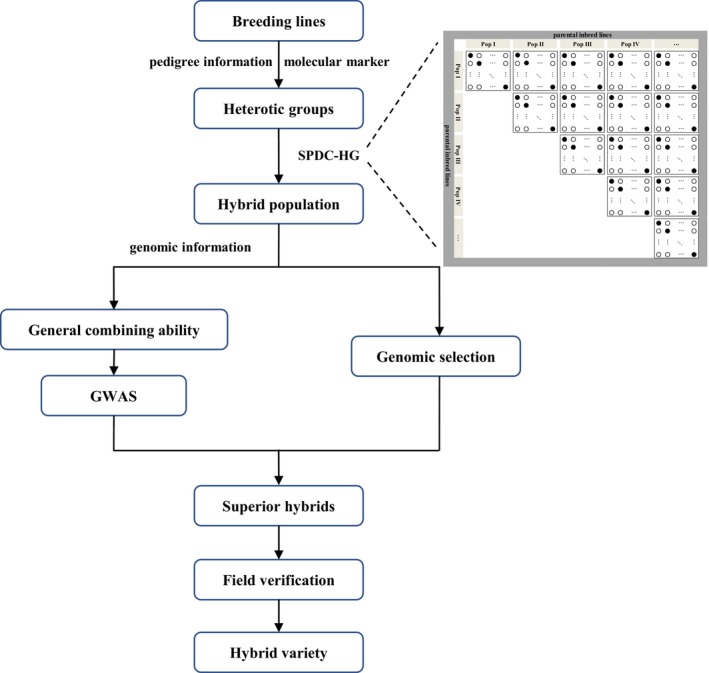
Genomic hybrid breeding roadmap based on SPDC‐HG.

The SPDC‐HG design used in this study was based on a collection of inbred lines from five HGs. These five HGs have been extensively employed in maize breeding in China, ensuring both genetic diversity and representativeness within the studied population. The GCA values for all 266 inbred lines were accurately assessed. The top‐ranked parents with the highest GCA values were selected, allowing for the potential evaluation of exceptional inbreds that have not yet been crossed within breeding pipelines. Notably, the estimated GCA values of the inbred lines associated with the top 100 and bottom 100 hybrids consistently ranked at the highest and lowest positions, respectively. This finding indicated that the GCA estimation was both effective and accurate. Furthermore, the flexible SPDC‐HG design demonstrated high efficiency in constructing populations for the simultaneous selection of hybrids and inbred lines. The GS strategy using the SPDC‐HG design offers breeders a practical and efficient experimental framework. This is expected to promote the accurate selection of hybrids and parental lines, which is crucial for the modern breeding of maize and other crops. Furthermore, we propose an efficient roadmap for genomic hybrid breeding based on SPDC‐HG (Figure 7). This roadmap incorporates a joint analysis of GWAS, GS and genomic evaluation of GCA in parental inbred lines, aiming to accelerate breeding progress in the development of single‐cross hybrids. Additionally, the robust field validation procedure provides a valuable reference for genomic hybrid breeding in maize.

The calculation of GCA constitutes a pivotal aspect of this study. Enhancing the calculation methodology of GCA is essential, particularly by incorporating genetic factors into the model, such as epistatic interactions and gene‐by‐environment (G × E) interactions. By accounting for epistatic interactions, the model can more accurately reflect the true genetic architecture of traits, leading to improved GCA calculations. Incorporating G × E interactions facilitates the sharing of information across correlated environments, and several studies have shown that GS models accounting for these interactions exhibit substantial improvements in predictability compared to models that consider only a single environment (Cuevas *et al*., 2016; Hu *et al*., 2023). Furthermore, the incorporation of downstream omics data, such as transcriptomic and metabolomic information, can capture interactions within and between various biological strata, thereby holding great potential to improve the accuracy of GCA calculations. Previous studies have demonstrated that the integration of multi‐omics data into GS has improved prediction accuracy for several crops (Schrag *et al*., 2018; Westhues *et al*., 2017; Xu *et al*., 2021b). Additionally, using advanced statistical models, such as deep learning, could be beneficial for estimating GCA. Deep learning models, with their multiple layers of non‐linear activation functions, possess advantages over traditional linear models by effectively capturing intricate relationships between variables, resulting in improved predictability (Wang *et al*., 2023). Overall, the integration of additional genetic factors, multi‐omics data and advanced statistical models presents a promising strategy to further improve GCA estimation in the future.

## Experimental procedures

### Plant materials and genotyping

A total of 266 elite inbred lines, including both temperate and tropical maize germplasms, were selected as parental lines for hybrid preparation. These lines are currently used for maize breeding in China. Genotyping‐by‐sequencing was applied to 266 inbred lines, and 199,152 SNPs across the entire maize genome were obtained. SNP quality control was performed by deleting SNPs with a missing rate > 20% and a minor allele frequency < 5%. Finally, 108,541 SNPs were identified for subsequent analysis. Neighbor‐joining (NJ) trees, Admixture and pedigree information were used to infer the population structure of the 266 inbred lines. A pairwise distance matrix derived from the simple matching distance for all SNPs was calculated to construct unweighted NJ trees using SNPhylo software (Lee *et al*., 2014), and phylogenetic trees were drawn using the online tool, iTOL (http://itol.embl.de/). Using Admixture, different levels of *K* (*K* = 2–15) were calculated to determine the optimum number of subpopulations based on the best K module. To verify the rationality of the subgroups, PCA was conducted using GCTA software (Yang *et al*., 2011).

### Development of the SPDC‐HG population and phenotyping

SPDC‐HG is a flexible and practical design for breeding programmes. It was mainly developed for producing single‐cross hybrids between inbred lines from different HGs. In this study, an SPDC‐HG scheme between five HGs of 266 inbred lines was performed during the winter of 2017 at an experimental station in Sanya, China. In 2018, 945 hybrids and 266 inbred lines were grown in two environments: Yangzhou (a spring maize growing area in China) and Taian (a summer maize growing area in China). A randomized block design with two replicates was used, with each hybrid planted in single‐row plots of 13 plants, 3 m long and 0.6 m between rows. Field management, including irrigation, fertilizer application and pest control, followed local agricultural practices. The PH (cm) and EH (cm) were measured before harvest. After maturity, five well‐developed ears from the middle of each plot were harvested by hand and air‐dried. EW (g), EGW (g), ERN, KNR, ED (cm), EL (cm) and CD were measured for each ear. For each plot, the average value for each trait was calculated for further analysis. The BLUP value of each material was used as the phenotypic value for further analysis.

### GWAS and LD analysis

In this study, five multi‐locus GWAS methods were used: Blink, FarmCPU, FASTmrMLM, FASTmrEMMA and ISIS EM‐BLASSO. The Blink and FarmCPU methods were implemented using the GAPIT software (Wang and Zhang, 2021), whilst FASTmrMLM, FASTmrEMMA and ISIS EM‐BLASSO were conducted using the R package mrMLM (Wen *et al*., 2017). Significant SNPs identified using the Blink and FarmCPU methods were screened with a threshold of −log_10_ (1/108541) = 5.036. A default LOD value of 3 was used as the threshold for screening significant SNPs identified by FASTmrMLM, FASTmrEMMA and ISIS EM‐BLASSO. Default values were applied for all other parameters. Manhattan plots were generated using the R package CMplot. LD for the 266 inbred lines was estimated using the squared correlation coefficient (*r* ^2^) between pairwise SNPs in PopLDdecay (Zhang *et al*., 2019).

### GS methods

GS was performed using three commonly used models, including GBLUP, BayesB and LASSO. GBLUP is a routine GS method that exploits the genomic relationships between the training and testing sets to predict the genomic values of unknown individuals (VanRaden, 2008). The GBLUP model was implemented using the R package predhy. BayesB employs a mixed distribution that includes a point of mass at zero and an inverted chi‐square distribution (Meuwissen *et al*., 2001). The BayesB model was fitted using the R/BGLR package with its default settings (Pérez and de los Campos, 2014). LASSO is a constrained form of ordinary least squares with a bound on the sum of the absolute values of the coefficients (Tibshirani, 1996). Here, LASSO was implemented using the R package glmnet (Friedman *et al*., 2010). The prediction accuracy is defined as the correlation coefficient between the observed and predicted phenotypic values, evaluated using fivefold cross‐validation with 20 repetitions.

### Accurate estimation of GCA

The linear mixed model for estimation of GCA is described as:

$$y=\mathrm{X\beta}+Z_{a}\gamma_{a}+Z_{d}\gamma_{d}+\varepsilon$$

where *γ* is an *n* × 1 vector of the phenotypic values; *X* is an design matrix for the fixed effect *β*; *Z*_*a*_ and *Z*_*d*_ are design matrices for the additive effect *γ*_*a*_ and dominance effect *γ*_*d*_, respectively and *ε* is a vector of residual errors with an assumed *N*(0,*Iσ* ^2^) distribution. It is assumed that $\gamma_{a}∼N\left(0,\frac{1}{m}\phi_{a}^{2}\right)$ and $\gamma_{d}\sim N\left(0,\frac{1}{m}\phi_{d}^{2}\right)$, where $\phi_{a}^{2}$ and $\phi_{d}^{2}$ are the corresponding polygenic variances, and *m* is the number of markers. The variance components were estimated using restricted maximum likelihood. After estimating the parameters from the training set, these can be used to predict the phenotypic values of the test set. Subsequently, the GCA for each line was estimated based on its average performance in hybrid combinations (Wang *et al*., 2020).

### Field validation

The EW values of the 945 hybrids were collected as the training set, and all potential 35,245 hybrids were predicted using BayesB, GBLUP and LASSO. The predicted EW values were sorted, and then crosses with the top 100 and bottom 100 EW values were selected. The selected single‐cross hybrids were generated during the winter of 2018 in Sanya, China. Subsequently, a field evaluation was performed on these hybrids during the spring maize growing season of 2019 in Yangzhou, China. Because of environmental and flowering factors, only parts of the hybrids were successfully observed in the field. These successfully crossed hybrids constituted the first field validation dataset (Hybrid_Set1). Unlike most previous studies that used correlation coefficients to evaluate GS effectiveness, this study evaluated GS by comparing the percentage increase in performance of the validated top 100 hybrids relative to the bottom 100 hybrids and a check variety. To analyze the role of significant SNPs identified through GWAS in predicting hybrid performance, 187 inbred lines were selected from the 266 inbred lines to generate hybrids, resulting in 259 hybrids that constituted the second validation dataset (Hybrid_Set2). A field evaluation of this dataset was performed during the spring maize growing season of 2020 in Yangzhou, China.

## Conflict of interest

The authors declare no conflict of interest.

## Author contributions

C.X. and Y.X. designed and supervised this research. Z.Z., Y.Z., K.Z., G.Y., W.Y., F.L. and X.G. performed experiments and collected data. Z.Z., X.W. and Z.Y. analysed data. Z.Z., X.W. and Y.X. wrote the manuscript. X.Z. assisted with the analysis and revised the manuscript. All the authors read and approved the manuscript.

## Supporting information


**Figure S1** Phenotypic variation of nine yield‐related traits categorized by different heterotic patterns.

**Figure S2** Prediction accuracies of nine yield‐related traits in hybrids using three GS models.

**Figure S3** Prediction accuracies of GCAs for nine yield‐related traits using three different models.

**Figure S4** Manhattan plots of nine yield‐related traits using five methods.

**Figure S5** Linkage disequilibrium decay distance of the 266 inbred lines.

**Figure S6** Cumulative effects of superior genotypes on GCAs. The horizontal axis (*n*) represents the number of superior genotypes and the vertical axis represents the GCA value of each trait. Linear regressions were performed to investigate the relationships between GCA and the number of superior genotypes.

**Figure S7** Cumulative effects of superior genotypes on phenotypes of hybrids. The horizontal axis (*n*) represents the number of superior genotypes and the vertical axis represents the phenotypes of hybrids for each trait. Linear regressions were performed to investigate the relationships between the phenotypes of hybrids and the number of superior genotypes.

**Figure S8** Accuracy of genomic prediction for 266 inbred lines with different numbers of inbred lines (*n* = 60, 150 or 266) involved in 500, 1000 and 1500 hybrids generated from the cross between those inbred lines.

**Table S1** Heterotic groups and names of the 266 selected inbred lines.

**Table S2** Detailed crossing scheme of the SPDC‐HG population.

**Table S3** Descriptive statistics and variance analysis of different heterotic patterns and all hybrids for the SPDC‐HG population.

**Table S4** BLUP values of the 945 hybrids.

**Table S5** Predicted EW values of the potential hybrids crossed amongst the 266 inbred lines using three methods.

**Table S6** The EW values of the 129 hybrids validated in the field.

**Table S7** Estimated GCA values for the 266 inbred lines.

**Table S8** Descriptive statistics for GCAs of the nine traits and multiple comparisons amongst different heterotic groups.

**Table S9** Significant SNPs of GCAs for nine yield‐related traits identified by five GWAS methods.

**Table S10** Significant SNPs of nine yield‐related traits identified by five GWAS methods.

**Table S11** Significant SNPs for GCAs detected by at least two statistical methods.

**Table S12** Significant SNPs for yield‐related traits detected by at least two statistical methods.

**Table S13** Prediction and annotation of candidate genes.

**Table S14** A novel hybrid population (Hybrid_Set2) generated from 187 inbred lines selected from the 266 inbred lines.

**Table S15** Analysis of favorable genotypes of significant GCA SNPs in inbred lines and hybrids.

## Data Availability

The data that supports the findings of this study are available in the supplementary material of this article.
