# Supplementary material for: SPDC‐HG: An accelerator of genomic hybrid breeding in maize
Source: Plant Biotechnol J. 2025 Feb 27;23(5):1847–61. doi: 10.1111/pbi.70011 (PMC12018846; doi:10.1111/pbi.70011)
Supplement: Supplementary file 1 — Figure S1 Phenotypic variation of nine yield‐related traits categorized by different heterotic patterns. [file PBI-23-1847-s007.docx]

**Figure S1** Phenotypic variation of nine yield-related traits categorized by different heterotic patterns.
