# Supplementary material for: SPDC‐HG: An accelerator of genomic hybrid breeding in maize
Source: Plant Biotechnol J. 2025 Feb 27;23(5):1847–61. doi: 10.1111/pbi.70011 (PMC12018846; doi:10.1111/pbi.70011)
Supplement: Supplementary file 2 — Figure S2 Prediction accuracies of nine yield‐related traits in hybrids using three GS models. [file PBI-23-1847-s018.docx]

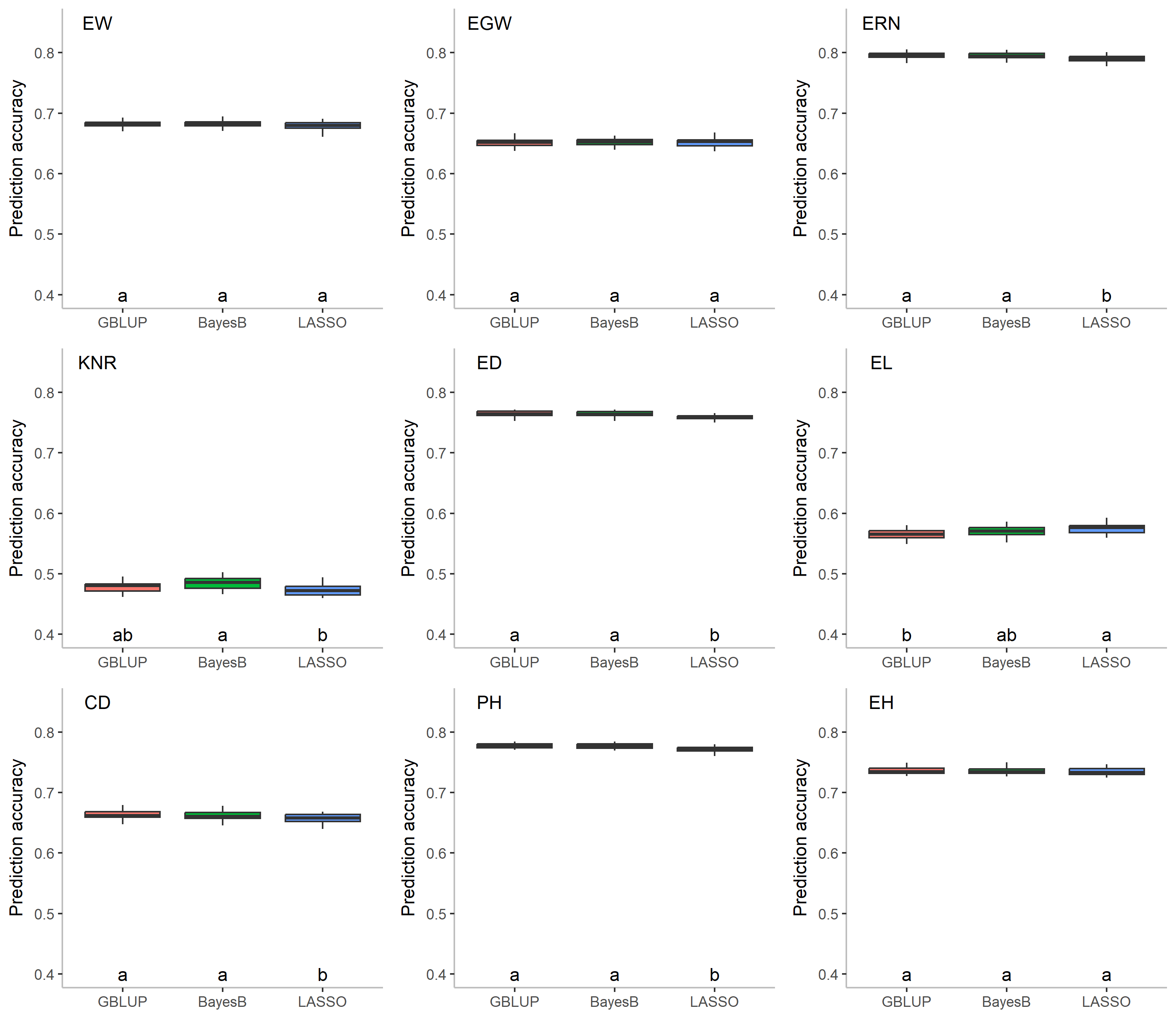


**Figure S2** Prediction accuracies of nine yield-related traits in hybrids using three GS models (letters indicated the significance levels (*P* < 0.05) of multiple comparisons).
