# Supplementary material for: SPDC‐HG: An accelerator of genomic hybrid breeding in maize
Source: Plant Biotechnol J. 2025 Feb 27;23(5):1847–61. doi: 10.1111/pbi.70011 (PMC12018846; doi:10.1111/pbi.70011)
Supplement: Supplementary file 3 — Figure S3 Prediction accuracies of GCAs for nine yield‐related traits using three different models. [file PBI-23-1847-s002.docx]

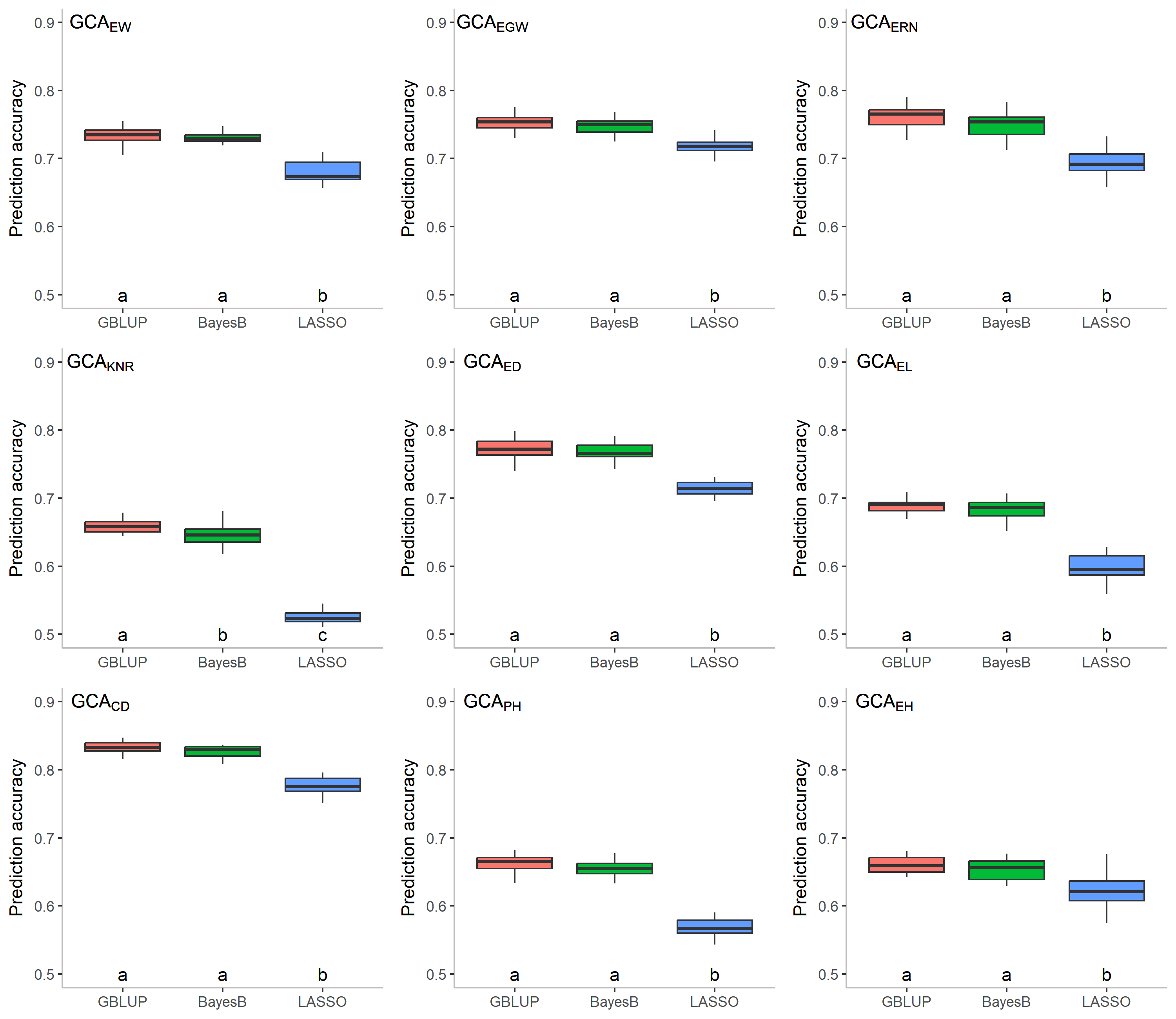


**Figure S3** Prediction accuracies of GCAs for nine yield-related traits using three different models (letters indicate the significance level of multiple comparisons).
