# Supplementary material for: SPDC‐HG: An accelerator of genomic hybrid breeding in maize
Source: Plant Biotechnol J. 2025 Feb 27;23(5):1847–61. doi: 10.1111/pbi.70011 (PMC12018846; doi:10.1111/pbi.70011)
Supplement: Supplementary file 4 — Figure S4 Manhattan plots of nine yield‐related traits using five methods. [file PBI-23-1847-s016.docx]

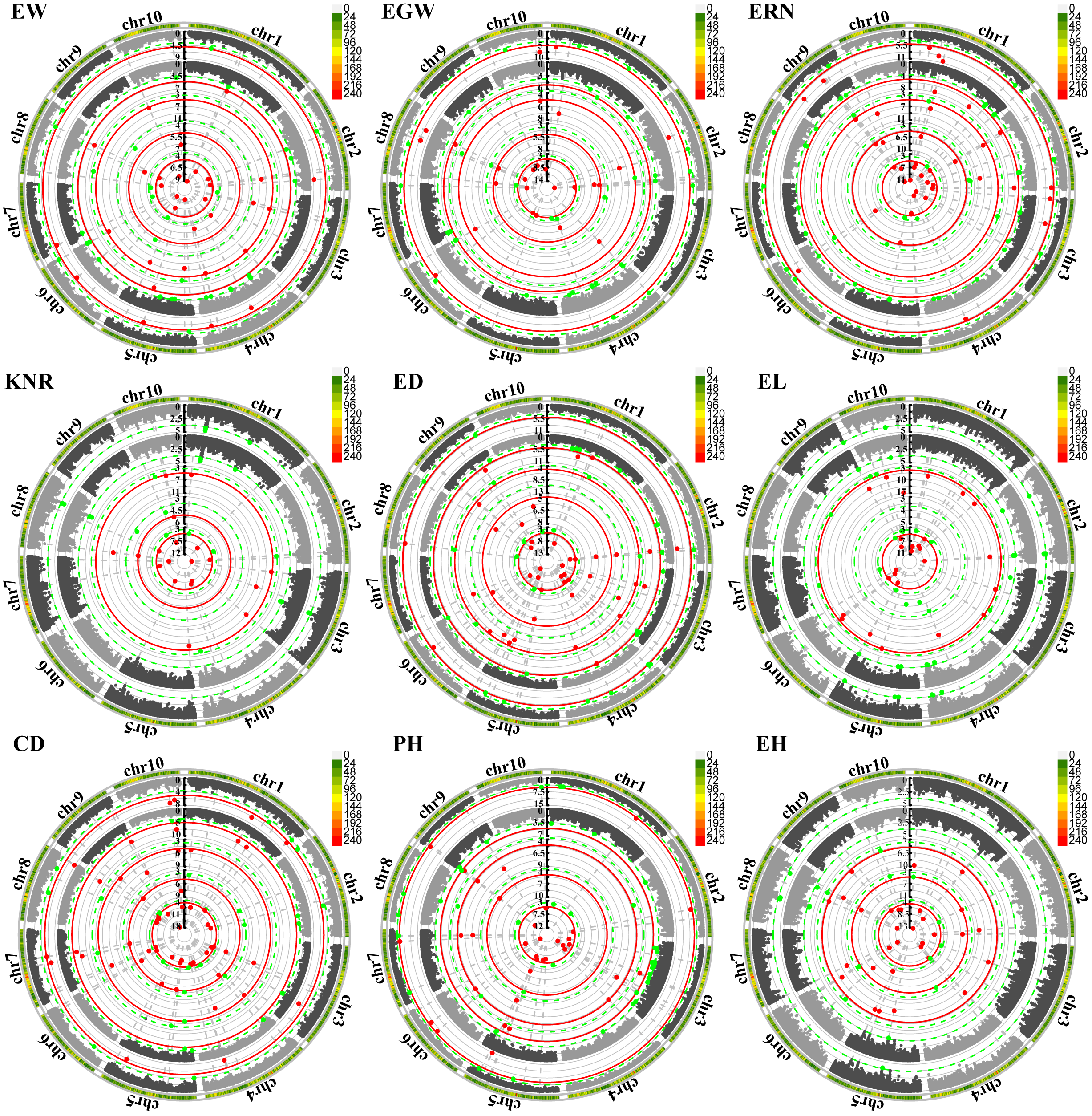


**Figure S4** Manhattan plots of nine yield-related traits using five GWAS methods. The outermost layer displays SNP marker density on chromosomes, with five y-axes showing results from Blink, FarmCPU, FASTmrMLM, FASTmrEMMA, and ISIS EM-BLASSO, respectively (from outside to inside). The red lines represent the threshold -log (1/108541) = 5.036, while the green dotted lines indicate the default LOD threshold of 3.
