# Supplementary material for: SPDC‐HG: An accelerator of genomic hybrid breeding in maize
Source: Plant Biotechnol J. 2025 Feb 27;23(5):1847–61. doi: 10.1111/pbi.70011 (PMC12018846; doi:10.1111/pbi.70011)
Supplement: Supplementary file 5 — Figure S5 Linkage disequilibrium decay distance of the 266 inbred lines. [file PBI-23-1847-s010.docx]

**Figure S5** Linkage disequilibrium decay distance of the 266 inbred lines
