# Supplementary material for: SPDC‐HG: An accelerator of genomic hybrid breeding in maize
Source: Plant Biotechnol J. 2025 Feb 27;23(5):1847–61. doi: 10.1111/pbi.70011 (PMC12018846; doi:10.1111/pbi.70011)
Supplement: Supplementary file 6 — Figure S6 Cumulative effects of superior genotypes on GCAs. The horizontal axis (n) represents the number of superior genotypes and the vertical axis represents the GCA value of each trait. Linear regressions were performed to investigate the relationships between GCA and the number of superior genotypes. [file PBI-23-1847-s001.docx]

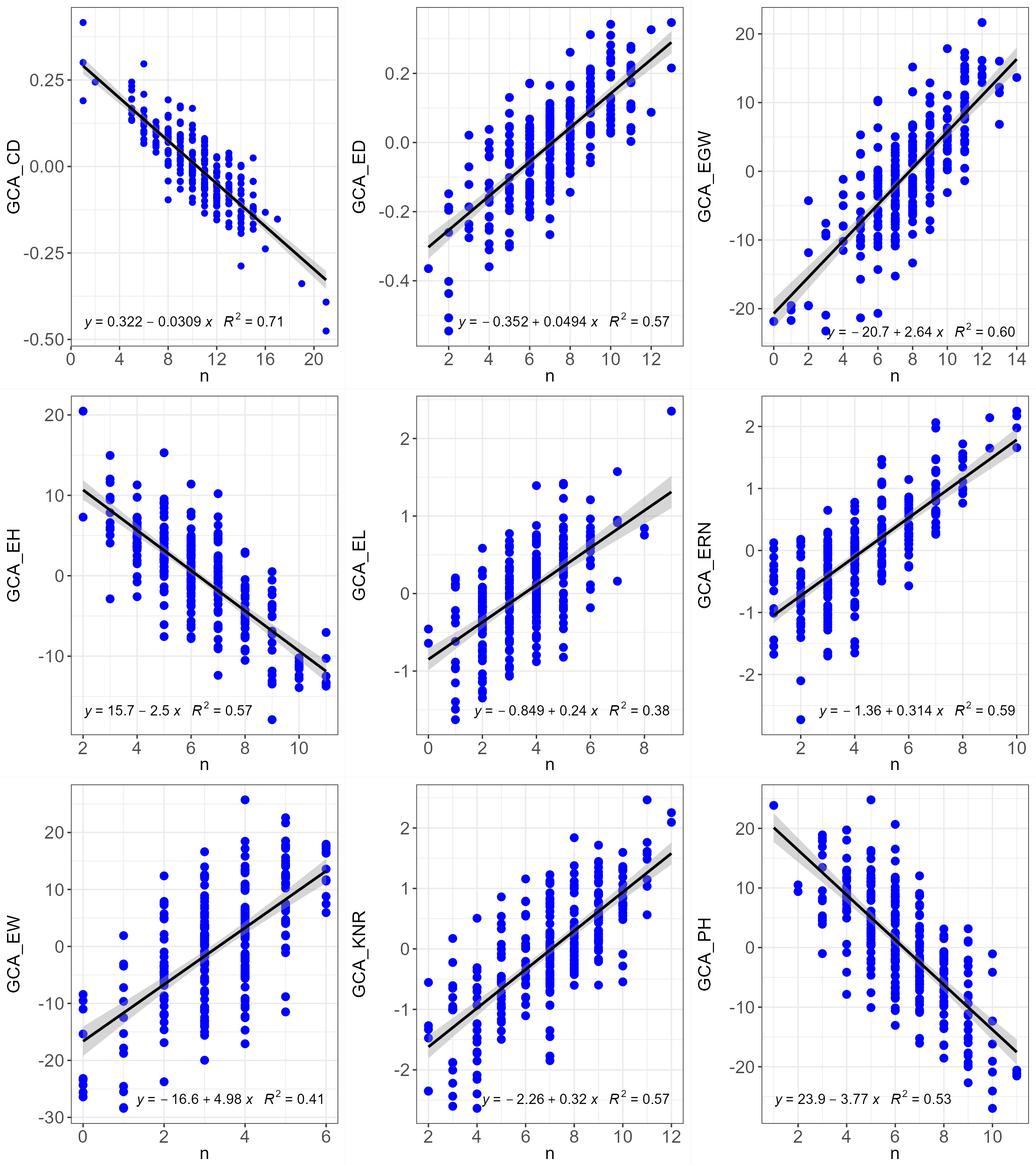


**Figure S6** Cumulative effects of superior genotypes on GCAs. The horizontal axis (n) represents the number of superior genotypes, and vertical axis represents the GCA value of each trait. Linear regressions were performed to investigate the relationships between GCA and number of superior genotypes.
