# Supplementary material for: SPDC‐HG: An accelerator of genomic hybrid breeding in maize
Source: Plant Biotechnol J. 2025 Feb 27;23(5):1847–61. doi: 10.1111/pbi.70011 (PMC12018846; doi:10.1111/pbi.70011)
Supplement: Supplementary file 7 — Figure S7 Cumulative effects of superior genotypes on phenotypes of hybrids. The horizontal axis (n) represents the number of superior genotypes and the vertical axis represents the phenotypes of hybrids for each trait. Linear regressions were performed to investigate the relationships between the phenotypes of hybrids and the number of superior genotypes. [file PBI-23-1847-s004.docx]

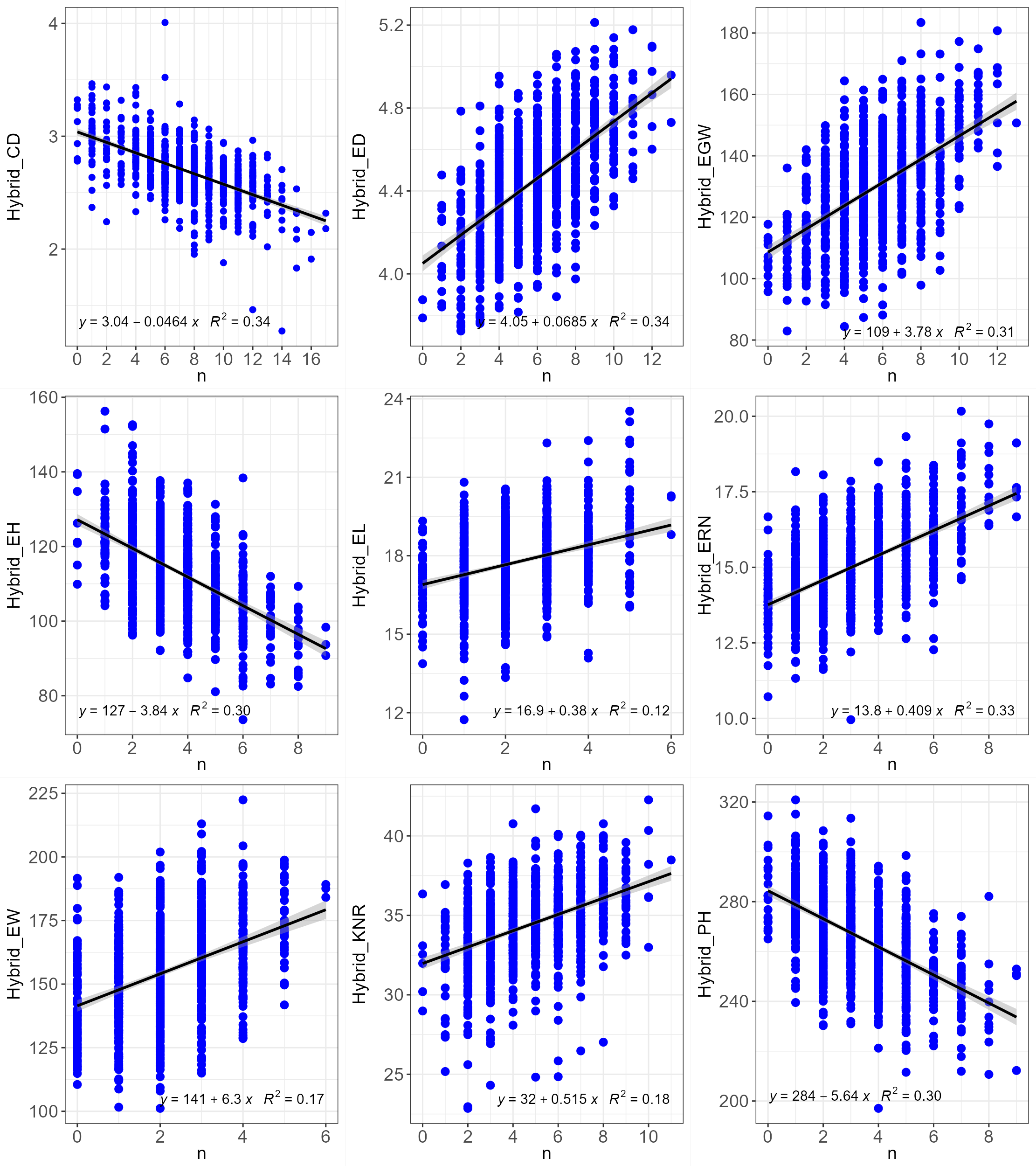


**Figure S7** Cumulative effects of superior genotypes on phenotypes of hybrids The horizontal axis (n) represents the number of superior genotypes, and vertical axis represents the phenotypes of hybrids for each trait. Linear regressions were performed to investigate the relationships between the phenotypes of hybrids and number of superior genotypes.
