# Supplementary material for: SPDC‐HG: An accelerator of genomic hybrid breeding in maize
Source: Plant Biotechnol J. 2025 Feb 27;23(5):1847–61. doi: 10.1111/pbi.70011 (PMC12018846; doi:10.1111/pbi.70011)
Supplement: Supplementary file 8 — Figure S8 Accuracy of genomic prediction for 266 inbred lines with different numbers of inbred lines (n = 60, 150 or 266) involved in 500, 1000 and 1500 hybrids generated from the cross between those inbred lines. [file PBI-23-1847-s013.docx]

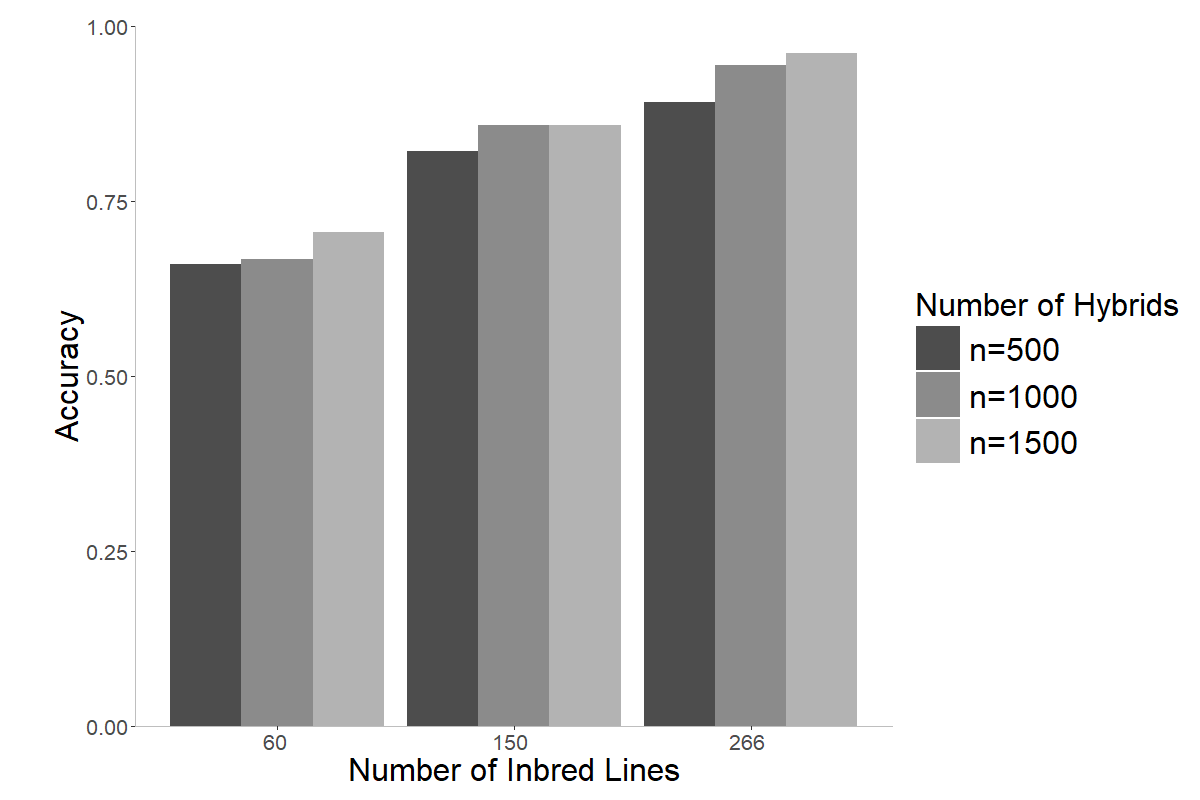


Figure S8 Accuracy of genomic prediction for 266 inbred lines with different numbers of inbred lines (n = 60, 150, or 266) involved in 500, 1000, and 1500 hybrids generated from the cross between those inbred lines.
